# Crystal structure of (*N* ^1^-benzyl-*N* ^1^,*N* ^2^,*N* ^2^-tri­methyl­ethane-1,2-di­amine-κ^2^ *N*,*N*′)di­chloridomercury(II)

**DOI:** 10.1107/S1600536814017516

**Published:** 2014-08-06

**Authors:** Sudesh T. Manjare, Harkesh B. Singh, Ray J. Butcher

**Affiliations:** a Department of Chemistry, Indian Institute of Technology Bombay, Powai, Mumbai 400 076, India; b Department of Chemistry, Howard University, 525 College Street NW, Washington, DC 20059, USA

**Keywords:** crystal structure, mercury complex, tertiary amine donors

## Abstract

In the structure of the title compound, [HgCl_2_(C_12_H_20_N_2_)], the Hg^II^ atom has a distorted tetra­hedral coordination sphere defined by two tertiary amine N-atom donors, as well as two Cl^−^ anions [the dihedral angle between the N—Hg—N and Cl—Hg—Cl planes is 82.80 (9)°]. The five-membered chelate ring adopts an envelope conformation, with puckering parameters of *Q*(2) = 0.446 (6) Å and ϕ(2) = 88.8 (6)°, with the two amine CH_3_ substituents on opposite sides of the ring. In the crystal, the mol­ecules are linked by C—H⋯Cl inter­actions into a zigzag chain parallel to [101].

## Abstract 2

The mol­ecular structure of [HgCl_2_(C_12_H_20_N_2_)] is a rare example where the Hg^II^ atom is bound to a Cl_2_N_2_ donor set for which the N atoms originate from aliphatic tertiary amine groups.

## Chemical context   

The chemistry of mercuric compounds with multidentate amine ligands is of inter­est due to the low coordination number and geometry preferences of Hg^II^, which facilitates extraordinarily rapid exchange of simple ligands (Bebout *et al.*, 2013 ▶; Carra *et al.*, 2013 ▶). The enhanced binding thermodynamics of these multidentate ligands has been used to suppress inter­molecular ligand-exchange rates for a variety of Hg^II^ complexes in solution, greatly enhancing the meaningfulness of NMR characterization. Significantly, under conditions of slow inter­molecular exchange the rates of intra­molecular isomerization processes for Hg^II^ can still exceed both the chemical shift and coupling constant time scale, particularly when bond cleavage is unnecessary and structures of these complexes have been determined (Bebout *et al.*, 2013 ▶; Carra *et al.*, 2013 ▶).
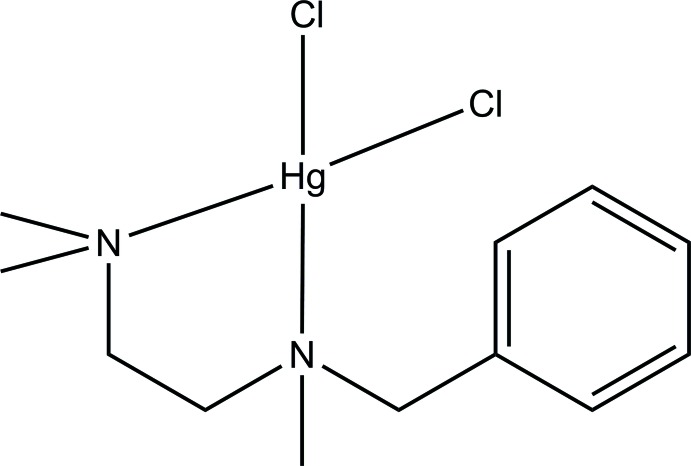


**Figure 1:**
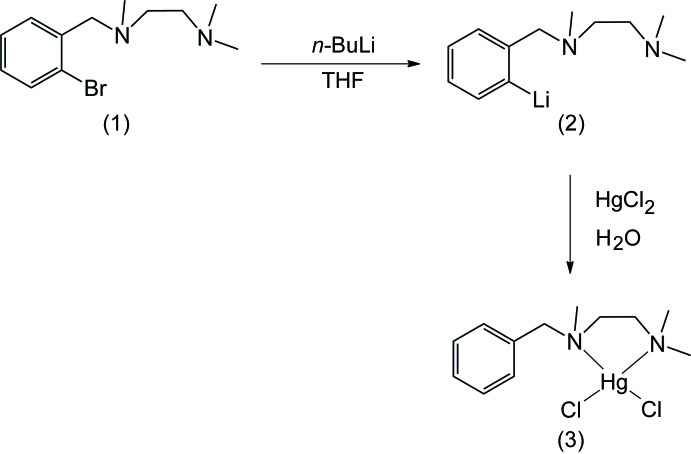
Reaction scheme showing the synthesis of the title compound.

In view of this inter­est in the coordination chemistry of mercury with multidentate amine ligands, and the lack of such structures involving tertiary amine donors, we report here the structure of the HgCl_2_ adduct of *N* ^1^-benzyl-*N* ^1^,*N* ^2^,*N* ^2^-tri­methyl­ethane-1,2-di­amine. The *o*-di­amine-substituted aryl bromide, *N* ^1^-(2-bromo­benz­yl)-*N* ^1^,*N* ^2^,*N* ^2^-tri­methyl­ethane-1,2-di­amine, can be prepared by the reaction of *N* ^1^,*N* ^1^,*N* ^2^-tri­methyl­ethane-1,2-di­amine and *ortho*-bromo­benzyl bromide. The ligand is moisture sensitive and is difficult to purify by column chromatography. However, it could easily be purified by vacuum distillation. The moisture-sensitive ligand, when treated with *n*-BuLi in tetra­hydro­furan (THF) and mercuric chloride, afforded the title compound, [HgCl_2_(C_12_H_20_N_2_)], (3) (Fig. 1 ▶).

## Structural commentary   

**Figure 2:**
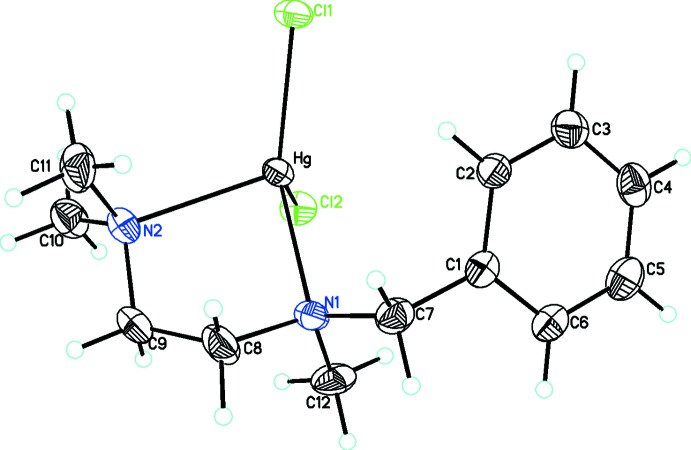
The mol­ecular structure of [HgCl_2_(C_12_H_20_N_2_)], showing the atom labelling and displacement ellipsoids at the 30% probability level.

In the structure of (3), the Hg^II^ atom is four-coordinated by two tertiary amine N-atom donors, as well as two Cl^−^ anions to give a distorted tetra­hedral coordination environment (Fig. 2 ▶). The distortion from ideal values can be seen by the dihedral angle between the N1—Hg—N2 and Cl1—Hg—Cl2 planes of 82.80 (9)°. The Hg—N and Hg—Cl bond lengths are in the normal ranges for such bonds (Allen, 2002 ▶). The five-membered chelate ring adopts an envelope conformation with puckering parameters of *Q*(2) = 0.446 (6)Å and ϕ(2) = 88.8 (6)° (Cremer & Pople, 1975 ▶), with the two amine CH_3_ substituents on opposite sides of the ring. Of the two reported structures which contain Hg^II^ attached to tertiary N donors (Choi *et al.*, 2005 ▶; Niu *et al.*, 2004 ▶), only one has Hg^II^ in an N_2_Cl_2_ coordination environment (Choi *et al.*, 2005 ▶) and thus provides the best comparison. The Hg—Cl [2.3875 (14) and 2.4397 (13) Å] and Hg—N bond lengths [2.355 (4) and 2.411 (4) Å] in (3) agree well with those found in the previous example [Hg—Cl = 2.397 (3) and 2.374 (2) Å; Hg—N = 2.353 (7) and 2.391 (6) Å].

## Supra­molecular features   

**Figure 3:**
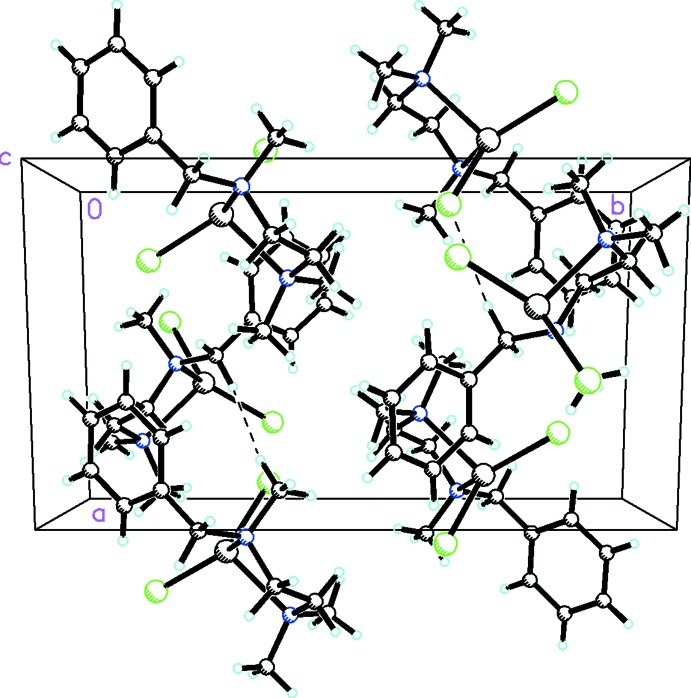
The mol­ecular packing for [HgCl_2_(C_12_H_20_N_2_)] viewed along the *c* axis. C—H⋯Cl inter­actions are shown as dashed lines.

The mol­ecular adducts are linked by C—H⋯Cl inter­actions (Table 1 ▶ and Fig. 3 ▶) into a zigzag chain parallel to [101]. As a result of the bulky nature of the complex, with the two amine CH_3_ substituents on opposite sides of the chelate ring, there is no evidence of any π–π inter­actions.

## Database survey   

In view of the inter­est in the coordination chemistry of mercury, it is surprising that a search of the Cambridge Structural Database (Version 5.35, November 2013 with one update; Allen, 2002 ▶) for structures of Hg^II^ with an N_2_Cl_2_ coordination sphere gave 96 hits, but the vast majority of these involved aromatic N donors such as pyridine and imidazole. There were only six hits involving aliphatic amine N-atom donors and only two (Choi *et al.*, 2005 ▶; Niu *et al.*, 2004 ▶) where the N atoms involved were both from tertiary amine functionalities.

## Synthesis and crystallization   

A stirred solution of *N* ^1^-(2-bromo­benz­yl)-*N* ^1^,*N* ^2^,*N* ^2^-tri­methyl­ethane-1,2-di­amine, (1), (1.10 ml, 5.34 mmol) in dry THF (15 ml) was treated dropwise with a 1.6 *M* solution of *n*-BuLi in hexane (3.80 ml, 6.15 mmol) *via* syringe under N_2_ at 273 K. On stirring the reaction mixture for 2 h at this temperature, the li­thia­ted product (2) was obtained. Mercuric chloride (1.55 g, 5.70 mmol) was added to the reaction mixture under a brisk flow of N_2_ gas and stirring was continued for an additional 6 h at room temperature. The reaction mixture was then removed from the N_2_ line and evaporated to dryness to give a colourless hygroscopic solid. The solid was extracted with dry chloro­form. The organic phase was separated, dried over Na_2_SO_4_, and filtered. The filtrate was evaporated to dryness to give a colourless crystalline solid of the HgCl_2_ adduct of *N* ^1^-benzyl-*N* ^1^,*N* ^2^,*N* ^2^-tri­methyl­ethane-1,2-di­amine, (3) (yield 1.25 g, 51%). The reaction scheme is shown in Fig. 1 ▶.

## Refinement   

Crystal data, data collection and structure refinement details are summarized in Table 2 ▶. H atoms were placed in geom­etric­ally idealized positions and constrained to ride on their parent atoms, with C—H distances of 0.95 (aromatic) and 0.99 Å (methyl­ene), with *U*_iso_(H) = 1.2*U*_eq_(C), and C—H = 0.98 Å for methyl H atoms, with *U*_iso_(H) = 1.5*U*_eq_(C).

## Supplementary Material

Crystal structure: contains datablock(s) 3. DOI: 10.1107/S1600536814017516/wm5031sup1.cif

Structure factors: contains datablock(s) 3. DOI: 10.1107/S1600536814017516/wm50313sup2.hkl

CCDC reference: 1016995

Additional supporting information:  crystallographic information; 3D view; checkCIF report

## Figures and Tables

**Table 1:** Hydrogen-bond geometry (Å, °)

*D*—H⋯*A*	*D*—H	H⋯*A*	*D*⋯*A*	*D*—H⋯*A*
C7—H7*A*⋯Cl2^i^	0.99	2.78	3.748 (6)	165

Symmetry code: (i) 
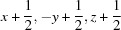
.

**Table 2:** Experimental details

Crystal data	
Chemical formula	[HgCl_2_(C_12_H_20_N_2_)]
*M*_r_	463.79
Crystal system, space group	Monoclinic, *P*2_1_/*n*
Temperature (K)	200
*a*, *b*, *c* (Å)	9.0839 (3), 15.5367 (6), 11.3161 (5)
β (°)	104.324 (4)
*V* (Å^3^)	1547.43 (10)
*Z*	4
Radiation type	Mo *K*α
μ (mm^−1^)	10.27
Crystal size (mm)	0.79 × 0.23 × 0.05

Data collection	
Diffractometer	Agilent Xcalibur
Absorption correction	Analytical [*CrysAlis PRO* (Agilent, 2014 ▶) using a multi-faceted crystal model based on expressions derived by Clark & Reid (1995 ▶)]
*T*_min_, *T*_max_	0.026, 0.339
No. of measured, independent and observed [*I* > 2σ(*I*)] reflections	13173, 5125, 3248
*R*_int_	0.067
(sin θ/λ)_max_ (Å^−1^)	0.758

Refinement	
*R*[*F* ^2^ > 2σ(*F* ^2^)], *wR*(*F* ^2^), *S*	0.044, 0.074, 0.96
No. of reflections	5125
No. of parameters	158
H-atom treatment	H-atom parameters constrained
Δρ_max_, Δρ_min_ (e Å^−3^)	1.54, −1.61

Computer programs: *CrysAlis PRO* (Agilent, 2014 ▶), *SHELXS97*, *SHELXL97* and *SHELXTL* (Sheldrick, 2008 ▶), *WinGX* (Farrugia, 2012 ▶) and *publCIF* (Westrip, 2010 ▶).

## supplementary crystallographic information

### Crystal data

**Table:**

[HgCl_2_(C_12_H_20_N_2_)]	*F*(000) = 880
*M**_r_* = 463.79	*D*_x_ = 1.991 Mg m^−^^3^
Monoclinic, *P*2_1_/*n*	Mo *K*α radiation, λ = 0.71073 Å
Hall symbol: -P 2yn	Cell parameters from 1518 reflections
*a* = 9.0839 (3) Å	θ = 5.3–30.8°
*b* = 15.5367 (6) Å	µ = 10.27 mm^−^^1^
*c* = 11.3161 (5) Å	*T* = 200 K
β = 104.324 (4)°	Plate, colorless
*V* = 1547.43 (10) Å^3^	0.79 × 0.23 × 0.05 mm
*Z* = 4	

### Data collection

**Table:**

Agilent Xcalibur diffractometer	5125 independent reflections
Radiation source: fine-focus sealed tube	3248 reflections with *I* > 2σ(*I*)
Graphite monochromator	*R*_int_ = 0.067
Detector resolution: 10.5081 pixels mm^-1^	θ_max_ = 32.6°, θ_min_ = 5.1°
ω scans	*h* = −13→11
Absorption correction: analytical [*CrysAlis PRO* (Agilent, 2014) using a multi-faceted crystal model based on expressions derived by Clark & Reid (1995)]	*k* = −16→23
*T*_min_ = 0.026, *T*_max_ = 0.339	*l* = −16→16
13173 measured reflections	

### Refinement

**Table:**

Refinement on *F*^2^	Secondary atom site location: difference Fourier map
Least-squares matrix: full	Hydrogen site location: inferred from neighbouring sites
*R*[*F*^2^ > 2σ(*F*^2^)] = 0.044	H-atom parameters constrained
*wR*(*F*^2^) = 0.074	*w* = 1/[σ^2^(*F*_o_^2^) + (0.0132*P*)^2^] where *P* = (*F*_o_^2^ + 2*F*_c_^2^)/3
*S* = 0.96	(Δ/σ)_max_ = 0.001
5125 reflections	Δρ_max_ = 1.54 e Å^−^^3^
158 parameters	Δρ_min_ = −1.61 e Å^−^^3^
0 restraints	Extinction correction: *SHELXL97* (Sheldrick, 2008), Fc^*^=kFc[1+0.001xFc^2^λ^3^/sin(2θ)]^-1/4^
Primary atom site location: structure-invariant direct methods	Extinction coefficient: 0.00248 (16)

### Special details

**Table:**

Geometry. All e.s.d.'s (except the e.s.d. in the dihedral angle between two l.s. planes) are estimated using the full covariance matrix. The cell e.s.d.'s are taken into account individually in the estimation of e.s.d.'s in distances, angles and torsion angles; correlations between e.s.d.'s in cell parameters are only used when they are defined by crystal symmetry. An approximate (isotropic) treatment of cell e.s.d.'s is used for estimating e.s.d.'s involving l.s. planes.
Refinement. Refinement of *F*^2^ against ALL reflections. The weighted *R*-factor *wR* and goodness of fit *S* are based on *F*^2^, conventional *R*-factors *R* are based on *F*, with *F* set to zero for negative *F*^2^. The threshold expression of *F*^2^ > σ(*F*^2^) is used only for calculating *R*-factors(gt) *etc*. and is not relevant to the choice of reflections for refinement. *R*-factors based on *F*^2^ are statistically about twice as large as those based on *F*, and *R*- factors based on ALL data will be even larger.

### Fractional atomic coordinates and isotropic or equivalent isotropic displacement parameters (Å^2^)

**Table:**

	*x*	*y*	*z*	*U*_iso_*/*U*_eq_	
Hg	0.11248 (2)	0.279066 (14)	0.55937 (2)	0.03718 (9)	
Cl1	0.24245 (15)	0.15445 (9)	0.51432 (13)	0.0476 (4)	
Cl2	−0.08589 (15)	0.34782 (10)	0.40263 (13)	0.0502 (4)	
N1	0.0504 (5)	0.3190 (3)	0.7420 (4)	0.0402 (11)	
N2	0.2991 (5)	0.3891 (3)	0.6352 (4)	0.0423 (11)	
C1	−0.0894 (5)	0.1816 (4)	0.7510 (5)	0.0368 (13)	
C2	−0.0412 (6)	0.1163 (4)	0.6876 (5)	0.0431 (14)	
H2A	0.0635	0.1125	0.6885	0.052*	
C3	−0.1412 (7)	0.0563 (4)	0.6228 (5)	0.0511 (16)	
H3A	−0.1059	0.0123	0.5784	0.061*	
C4	−0.2928 (7)	0.0605 (4)	0.6231 (5)	0.0531 (16)	
H4A	−0.3624	0.0194	0.5788	0.064*	
C5	−0.3428 (6)	0.1239 (4)	0.6871 (6)	0.0552 (17)	
H5A	−0.4470	0.1263	0.6879	0.066*	
C6	−0.2417 (6)	0.1853 (4)	0.7516 (5)	0.0481 (15)	
H6A	−0.2773	0.2294	0.7957	0.058*	
C7	0.0218 (6)	0.2459 (4)	0.8187 (5)	0.0455 (14)	
H7A	0.1193	0.2163	0.8541	0.055*	
H7B	−0.0163	0.2691	0.8870	0.055*	
C8	0.1829 (7)	0.3687 (4)	0.8088 (6)	0.0572 (18)	
H8A	0.2647	0.3280	0.8471	0.069*	
H8B	0.1540	0.4009	0.8752	0.069*	
C9	0.2449 (7)	0.4314 (4)	0.7321 (6)	0.0574 (17)	
H9A	0.1644	0.4732	0.6950	0.069*	
H9B	0.3298	0.4638	0.7852	0.069*	
C10	0.3076 (7)	0.4511 (4)	0.5401 (6)	0.0597 (18)	
H10A	0.3811	0.4962	0.5747	0.090*	
H10B	0.2074	0.4770	0.5077	0.090*	
H10C	0.3401	0.4216	0.4743	0.090*	
C11	0.4498 (6)	0.3478 (4)	0.6837 (7)	0.064 (2)	
H11A	0.5219	0.3906	0.7281	0.095*	
H11B	0.4871	0.3247	0.6158	0.095*	
H11C	0.4398	0.3008	0.7390	0.095*	
C12	−0.0886 (6)	0.3744 (4)	0.7088 (6)	0.0575 (17)	
H12A	−0.1164	0.3928	0.7833	0.086*	
H12B	−0.1725	0.3415	0.6575	0.086*	
H12C	−0.0679	0.4251	0.6641	0.086*	

### Atomic displacement parameters (Å^2^)

**Table:**

	*U*^11^	*U*^22^	*U*^33^	*U*^12^	*U*^13^	*U*^23^
Hg	0.03850 (13)	0.03097 (13)	0.04205 (14)	−0.00121 (10)	0.00990 (8)	−0.00793 (11)
Cl1	0.0585 (9)	0.0360 (8)	0.0541 (9)	0.0049 (7)	0.0249 (7)	−0.0088 (7)
Cl2	0.0470 (8)	0.0549 (10)	0.0448 (9)	0.0077 (7)	0.0037 (6)	−0.0001 (8)
N1	0.050 (3)	0.032 (3)	0.041 (3)	−0.001 (2)	0.017 (2)	−0.006 (2)
N2	0.047 (3)	0.031 (3)	0.046 (3)	−0.004 (2)	0.007 (2)	−0.001 (2)
C1	0.034 (3)	0.039 (3)	0.037 (3)	0.007 (2)	0.008 (2)	0.012 (3)
C2	0.044 (3)	0.033 (3)	0.057 (4)	0.008 (3)	0.021 (3)	0.010 (3)
C3	0.069 (4)	0.040 (4)	0.048 (4)	0.003 (3)	0.022 (3)	0.009 (3)
C4	0.068 (4)	0.050 (4)	0.039 (4)	−0.013 (3)	0.009 (3)	0.009 (3)
C5	0.038 (3)	0.072 (5)	0.055 (4)	0.002 (3)	0.009 (3)	0.010 (4)
C6	0.046 (3)	0.055 (4)	0.047 (4)	0.003 (3)	0.018 (3)	0.006 (3)
C7	0.051 (3)	0.045 (4)	0.045 (4)	0.006 (3)	0.020 (3)	0.006 (3)
C8	0.076 (4)	0.050 (4)	0.046 (4)	−0.023 (3)	0.015 (3)	−0.018 (3)
C9	0.068 (4)	0.042 (4)	0.064 (4)	−0.018 (3)	0.020 (3)	−0.021 (3)
C10	0.075 (4)	0.043 (4)	0.061 (4)	−0.008 (3)	0.018 (3)	−0.001 (3)
C11	0.035 (3)	0.058 (5)	0.089 (5)	−0.006 (3)	−0.001 (3)	0.000 (4)
C12	0.062 (4)	0.048 (4)	0.069 (5)	0.026 (3)	0.029 (3)	0.003 (3)

### Geometric parameters (Å, º)

**Table:**

Hg—N1	2.355 (4)	C5—C6	1.398 (8)
Hg—Cl1	2.3875 (14)	C5—H5A	0.9500
Hg—N2	2.411 (4)	C6—H6A	0.9500
Hg—Cl2	2.4397 (13)	C7—H7A	0.9900
N1—C8	1.472 (6)	C7—H7B	0.9900
N1—C7	1.491 (7)	C8—C9	1.503 (9)
N1—C12	1.497 (6)	C8—H8A	0.9900
N2—C10	1.460 (7)	C8—H8B	0.9900
N2—C9	1.465 (8)	C9—H9A	0.9900
N2—C11	1.489 (7)	C9—H9B	0.9900
C1—C2	1.375 (7)	C10—H10A	0.9800
C1—C6	1.386 (7)	C10—H10B	0.9800
C1—C7	1.491 (8)	C10—H10C	0.9800
C2—C3	1.379 (8)	C11—H11A	0.9800
C2—H2A	0.9500	C11—H11B	0.9800
C3—C4	1.380 (8)	C11—H11C	0.9800
C3—H3A	0.9500	C12—H12A	0.9800
C4—C5	1.364 (8)	C12—H12B	0.9800
C4—H4A	0.9500	C12—H12C	0.9800
			
N1—Hg—Cl1	129.73 (12)	C1—C7—N1	113.9 (5)
N1—Hg—N2	78.51 (16)	C1—C7—H7A	108.8
Cl1—Hg—N2	108.04 (12)	N1—C7—H7A	108.8
N1—Hg—Cl2	103.21 (11)	C1—C7—H7B	108.8
Cl1—Hg—Cl2	121.01 (5)	N1—C7—H7B	108.8
N2—Hg—Cl2	106.03 (11)	H7A—C7—H7B	107.7
C8—N1—C7	109.8 (4)	N1—C8—C9	114.7 (5)
C8—N1—C12	111.0 (5)	N1—C8—H8A	108.6
C7—N1—C12	109.0 (4)	C9—C8—H8A	108.6
C8—N1—Hg	104.3 (3)	N1—C8—H8B	108.6
C7—N1—Hg	115.1 (3)	C9—C8—H8B	108.6
C12—N1—Hg	107.6 (3)	H8A—C8—H8B	107.6
C10—N2—C9	110.1 (5)	N2—C9—C8	112.6 (5)
C10—N2—C11	110.1 (5)	N2—C9—H9A	109.1
C9—N2—C11	111.5 (5)	C8—C9—H9A	109.1
C10—N2—Hg	111.5 (3)	N2—C9—H9B	109.1
C9—N2—Hg	104.4 (3)	C8—C9—H9B	109.1
C11—N2—Hg	109.1 (3)	H9A—C9—H9B	107.8
C2—C1—C6	118.7 (5)	N2—C10—H10A	109.5
C2—C1—C7	119.9 (5)	N2—C10—H10B	109.5
C6—C1—C7	121.3 (5)	H10A—C10—H10B	109.5
C1—C2—C3	121.5 (5)	N2—C10—H10C	109.5
C1—C2—H2A	119.2	H10A—C10—H10C	109.5
C3—C2—H2A	119.2	H10B—C10—H10C	109.5
C2—C3—C4	119.5 (6)	N2—C11—H11A	109.5
C2—C3—H3A	120.2	N2—C11—H11B	109.5
C4—C3—H3A	120.2	H11A—C11—H11B	109.5
C5—C4—C3	119.9 (6)	N2—C11—H11C	109.5
C5—C4—H4A	120.0	H11A—C11—H11C	109.5
C3—C4—H4A	120.0	H11B—C11—H11C	109.5
C4—C5—C6	120.5 (6)	N1—C12—H12A	109.5
C4—C5—H5A	119.7	N1—C12—H12B	109.5
C6—C5—H5A	119.7	H12A—C12—H12B	109.5
C1—C6—C5	119.8 (6)	N1—C12—H12C	109.5
C1—C6—H6A	120.1	H12A—C12—H12C	109.5
C5—C6—H6A	120.1	H12B—C12—H12C	109.5
			
Cl1—Hg—N1—C8	89.5 (4)	C7—C1—C2—C3	−179.3 (5)
N2—Hg—N1—C8	−14.5 (4)	C1—C2—C3—C4	−1.2 (9)
Cl2—Hg—N1—C8	−118.5 (3)	C2—C3—C4—C5	0.0 (9)
Cl1—Hg—N1—C7	−30.9 (4)	C3—C4—C5—C6	0.7 (9)
N2—Hg—N1—C7	−134.9 (4)	C2—C1—C6—C5	−0.9 (8)
Cl2—Hg—N1—C7	121.2 (3)	C7—C1—C6—C5	−179.9 (5)
Cl1—Hg—N1—C12	−152.6 (3)	C4—C5—C6—C1	−0.3 (9)
N2—Hg—N1—C12	103.4 (4)	C2—C1—C7—N1	85.1 (6)
Cl2—Hg—N1—C12	−0.6 (4)	C6—C1—C7—N1	−95.8 (6)
N1—Hg—N2—C10	−132.3 (4)	C8—N1—C7—C1	−168.4 (5)
Cl1—Hg—N2—C10	99.4 (4)	C12—N1—C7—C1	69.8 (6)
Cl2—Hg—N2—C10	−31.7 (4)	Hg—N1—C7—C1	−51.1 (5)
N1—Hg—N2—C9	−13.4 (4)	C7—N1—C8—C9	166.8 (5)
Cl1—Hg—N2—C9	−141.7 (3)	C12—N1—C8—C9	−72.6 (7)
Cl2—Hg—N2—C9	87.2 (4)	Hg—N1—C8—C9	43.0 (6)
N1—Hg—N2—C11	105.9 (4)	C10—N2—C9—C8	160.3 (5)
Cl1—Hg—N2—C11	−22.4 (4)	C11—N2—C9—C8	−77.1 (6)
Cl2—Hg—N2—C11	−153.5 (4)	Hg—N2—C9—C8	40.5 (6)
C6—C1—C2—C3	1.6 (8)	N1—C8—C9—N2	−61.6 (7)

### Hydrogen-bond geometry (Å, º)

**Table:**

*D*—H···*A*	*D*—H	H···*A*	*D*···*A*	*D*—H···*A*
C7—H7*A*···Cl2^i^	0.99	2.78	3.748 (6)	165

Symmetry code: (i) *x*+1/2, −*y*+1/2, *z*+1/2.
